# Differentiating cerebral ischemia from functional neurological symptom disorder: a psychosomatic perspective

**DOI:** 10.1186/1471-244X-14-158

**Published:** 2014-05-29

**Authors:** Carl E Scheidt, Kathrin Baumann, Michael Katzev, Matthias Reinhard, Sebastian Rauer, Michael Wirsching, Andreas Joos

**Affiliations:** 1 Department of Psychosomatic Medicine and Psychotherapy, University of Freiburg, Hauptstraße 8, D - 79104 Freiburg, Germany; 2 Department of Neurology, University of Freiburg, Breisacher Straße 64, D - 79106 Freiburg, Germany

**Keywords:** Conversion disorder, Functional neurological symptom disorder, Psychosomatic medicine, DSM-5, ICD-11

## Abstract

**Background:**

The differential diagnosis of pseudo-neurological symptoms often represents a clinical challenge. The Diagnostic and Statistical Manual of Mental Disorders, DSM-5, made an attempt to improve diagnostic criteria of conversion disorder (functional neurological symptom disorder). Incongruences of the neurological examination, i.e. positive neurological signs, indicate a new approach - whereas psychological factors are not necessary anymore. As the DSM-5 will influence the International Classification of Diseases, ICD-11, this is of importance. In the case presented, a history of psychological distress and adverse childhood experiences coexisted with a true neurological disorder. We discuss the relevance of an interdisciplinary assessment and of operationalized diagnostic criteria.

**Case presentation:**

A 32-year-old man presented twice with neurological symptoms without obvious pathological organic findings. A conversion disorder was considered early on at the second admission by the neurology team. Sticking to ICD-10, this diagnosis was not supported by a specialist for psychosomatic medicine, due to missing hints of concurrent psychological distress in temporal association with neurological symptoms. Further investigations then revealed a deep vein thrombosis (though D-dimers had been negative), which had probably resulted in a crossed embolus.

**Conclusion:**

The absence of a clear proof of biological dysfunction underlying neurological symptoms should not lead automatically to the diagnosis of a conversion disorder. In contrast, at least in more complex patients, the work-up should include repeated psychological and neurological assessments in close collaboration. According to ICD-10 positive signs of concurrent psychological distress are required, while DSM-5 emphasizes an incongruity between neurological symptoms and neurophysiological patterns of dysfunction. In the case presented, an extensive medical work-up was initially negative, and neither positive psychological nor positive neurological criteria could be identified. We conclude, that, even in times of more sophisticated operationalization of diagnostic criteria, the interdisciplinary assessment has to be based on an individual evaluation of all neurological and psychosocial findings. Prospective studies of inter-rater reliability and validity of psychological factors and positive neurological signs are needed, as evidence for both is limited. With respect to ICD-11, we suggest that positive neurological as well as psychological signs for functional neurological symptom disorder should be considered to increase diagnostic certainty.

## Background

Conversion disorder or functional neurological symptom disorder (terms which we will use interchangeably) is common in clinical practice, making up one third of neurology outpatients and approximately 9% of inpatients [1,2]. The disorder falls increasingly somewhere between neurology and psychiatry (for details see: [2,3]). In many cases a close collaboration between neurology and psychosomatic medicine with repeated mutual consultations is necessary. This accounts for the diagnostic work-up as well as for treatment. Through this case presentation, we will demonstrate the value of a close collaboration, i.e. a psychosomatic liaison service, and discuss some aspects of the transition from the fourth to the fifth edition of the Diagnostic and Statistical Manual of Mental Disorders, i.e. DSM-IV [4] to DSM-5 [5]. This is of particular importance as the International Classification of Diseases, ICD-10 [6], which is currently in world-wide use, is also being revised, and ICD-11 is due in about 2015/16.

DSM-5 [5] introduced profound and partially controversial changes of diagnostic criteria of various disorders [7-9]. With respect to functional neurological symptom disorder, DSM-5 reduced the number of diagnostic criteria from six to four. On the content level, Criterion (A) (sensory or motor symptoms) and (D) (symptoms causing psychosocial distress) seem relatively vague and are hardly operationalized. These criteria may account for many neurological presentations in daily practice - regardless of their cause. Criterion (C) implies negative medical/organic findings. Criterion (B) is new and relates to “positive neurological signs”, which will be considered below. Instead, the presence of psychological factors associated with the physical symptoms (in contrast to ICD-10 and DSM-IV) is no longer required.

In this case report we will emphasize the impact of a comprehensive and ongoing *psychosomatic and neurological diagnostic assessment* of conversion disorder. Concerning the current diagnostic manuals and their transition, i.e. DSM and ICD [4-6] we will consider three issues in the subsequent paragraphs. Furthermore, we will discuss the necessity of a thorough biopsychosocial assessment, which is of particular importance, because these patients are often (not always) reluctant to consider psychosocial aspects related to their complaints by themselves [3].

1. There have been major advances in diagnostic tools in neurology, in particular neuroimaging, allowing to detect even very small cerebral lesions [10]. Nowadays, magnetic resonance imaging (MRI) is widely used in routine clinical practice. Therefore, neurologic work-up has become quite accurate and decisive, leading to rapid treatment recommendations (e.g. in stroke or multiple sclerosis). Only a very small proportion of patients initially presenting with organically unexplained symptoms will show organic etiologies at hindsight, and the rate has decreased significantly over the last decades [1,3,11]. Therefore, the authority of the initial neurological work-up is substantial, and negative findings rapidly reinforce a consideration of functional neurological symptom disorder as differential diagnosis [3]. In DSM-5, criterion (C) alludes to negative medical findings. (DSM IV and ICD-10 also require this criterion.)

2. It has long been recognized that it is difficult to reliably operationalize complex psychological factors such as conflicts, stress or trauma, as diagnostic criteria for conversion disorder at the time of onset of symptoms [12-14]. Though stressful life events and active coping difficulties are more common in such patients [11,15], there are no common and empirically validated tests [3,16]. In fact, findings like *la belle indifference* are no more common in subjects with functional disorders than in other diagnostic groups [17]. Furthermore, patients are often reluctant to accept psychological associations [3] including stress [18]. In consequence, DSM-5 abandoned psychological criteria, and uses psychological stressors as specifier only [5]. The accompanying text in DSM-5 does not refer to psychological aspects in detail. The DSM-IV approach describing that the *“individual's somatic symptom represents a symbolic resolution of an unconscious psychological conflict…”*[4] and referring to psychodynamic constructs was abandoned.

Considering the development of DSM-5, the transition from ICD-10 [6] to ICD-11 with respect to criteria and nomenclature of functional neurological symptom disorder will be most interesting - since the ICD-10 just as DSM-IV relies on a psychological formulation of diagnostic criteria [6].

3. A new approach is presented in DSM-5 by Criterion (B), which concurs with an “incompatibility between the symptom and recognized neurological or medical conditions” [5], i.e. “positive evidence of internal inconsistency or incongruity with disease” [14]. This new way of thinking is a major advance since the task of neurology is no longer limited to the exclusion of an organic etiology, but is also to find positive clues leading to a correct diagnosis. However - as with psychological factors – empirical evidence is limited and focusing on motor signs [11,19]. Sensitivity and positive predictive values for these signs vary, and they are low for some [19]. Hence, negative findings have limitations in the diagnostic process. In addition and of practical relevance: These signs and concepts do not yet constitute a substantial part of current neurological training. In effect, in the latest edition of our neurology textbook we emphasize these signs and commit a whole section to this topic [20]. DSM-5 emphasizes this approach in the accompanying text and mentions singular specific positive neurological signs of conversion (e.g. Hoover’s sign) - but abstains to use these directly in order to fulfill criterion (B), i.e. to operationalize more specifically.

Stroke - which is the differential diagnosis in the case reported here - is one of the major causes of morbidity and mortality in Western society. While atherothrombosis and atrial fibrillation are prominent causes for ischemic stroke in older subjects, in younger patients the spectrum is much wider, including cerebral artery dissection, clotting defects and patent foramen ovale. Still, 25–30% of ischemic strokes are classified as “cryptogenic” [21]. Depending on clinical presentation, functional neurological symptom disorder, which accounts for up to 30% of neurological outpatient visits [1], has to be taken into account as differential diagnosis in patients admitted with signs of acute stroke.

In the case reported here, initially there were no definite medical findings for validating the diagnosis of ischemic stroke. Conversion was considered as differential diagnosis. However, there were neither positive neurological nor psychological signs. Only an ongoing discussion of the case between neurologists and psychosomatic specialists and a stepwise extension of laboratory work-up led to the correct diagnosis. One step in this process was the rejection of suspected functional neurological symptom disorder because of the absence of conflict and stress. In other words, the presence of positive psychological signs related to the neurological symptoms could not be substantiated - which is a necessary criterion for the diagnosis of conversion disorder according to ICD-10.

In reporting this case we want to illustrate the necessity of a close clinical cooperation of neurologists and psychosomatic specialists in the diagnostic work-up of neurological and pseudo-neurological symptoms. In addition, we want to contribute some thoughts on the usefulness of positive psychological and neurological criteria for the differential diagnosis of functional neurological symptoms.

## Case presentation

A 32-year-old man initially presented with a mild sensory-motor paralysis of the right side. Extensive neurological work-up, including MRI, electrophysiology (somatosensory and motor potentials, electroencephalography), ultrasound of cerebral arteries, and laboratory findings including examination of cerebrospinal fluid were normal. Transesophageal echocardiography had shown mild contrast medium flow from the right to the left atrium under Valsalva maneuver without demonstration of an atrial septal aneurysm (due to strong retching further description was not possible). At discharge 11 days later, a very mild paresis of the right arm was described. The patient was discharged with a suspected brainstem ischemia and a suspected open foramen ovale. He was started on Aspirin 100 mg/day.

Three months later he returned to the emergency department with transiently worsened right-sided sensory-motor hemiparesis and in addition transient speech problems (mainly consisting of difficulties of his wife understanding him, which he was not able to describe in more detail retrospectively). The transient speech problems could not be categorized definitely, but seemed more likely of a dysphonic/dysarthric nature. Though it is of importance to distinguish between dysphasia and dysarthria, this can be a difficult and sometimes impossible task in transient symptoms [22]. The neurological examination at admission was unremarkable except for minor hypoesthesia of the right arm. No other neurological signs were revealed, and there was no evidence for internal inconsistency of the neurological examination, i.e. positive neurological signs. Again, additional laboratory work-up including MR-angiography gave no hints for the etiology. At this second admission, the psychosomatic liaison service was contacted very early on by the neurology team with the question of a possible conversion disorder. Furthermore, the patient was distressed by the re-occurrence of his neurological deficits. Therefore, the task of the psychosomatic physician was to support him during his hospital stay, in addition to evaluating diagnostic issues.

The patient was married, had two children and was working fulltime as a shift leader in a cement plant. His biography showed several strains, e.g. an alcohol dependent father. Due to strong conflicts with his family of origin he had moved to an aunt at age 16. His life had been devoted to an excessive amount of work. Prior to his first admission, the work situation had been heavily strained due to illnesses of co-workers. In talking to the psychosomatic physician, he pondered that all his life he has had great difficulties in rejecting requests of others. His psychosocial activities and his family life however seemed rather intact, and he had not attended to psychological treatment before. He was very irritated by his neurological symptoms, in particular as there had not been a definite cause during his first stay. Our board-certified psychosomatic consultant (KB) considered the work-related strains prior to the first admission as unspecific. Further she concluded that there was not sufficient evidence of conflict or stress in temporal association with the current neurologic symptomatology at the second admission - although there were obvious strains in his earlier biography. Sticking to ICD-10 and its diagnostic criteria, she abstained from supporting the diagnosis of functional neurological symptom disorder. Furthermore, there was no associated affective co-morbidity justifying an ICD-diagnosis (e.g. adjustment disorder). In effect, no further psychotherapeutic intervention after the hospital stay was recommended.

The case was discussed with the neurology team extensively and further evaluation followed immediately. As a next step, ultrasound of the right leg was carried out due to a minor ache in his right calf ad admission – even though D-dimers had been negative initially. This proved a deep vein thrombosis above knee (into the femoral vein), which had probably resulted in a paradoxical embolism. The patient was discharged on oral anticoagulation.

## Discussion

This case demonstrates that the differential diagnosis of functional neurological symptom disorders is a complex and an interdisciplinary task [3], which often has to be done as a continuing process: In the case reported after a first suspicion by the neurology team, the psychosomatic specialist was reluctant to support the diagnosis, and the search on the side of the neurologists continued. Therefore, in all complex cases, the diagnostic assessment is not a “yes - no decision”, but rather a *process of integrating neurological and psychosocial findings and perspectives*. Like in this case, treatment recommendations vary dramatically depending on diagnosis (e.g. psychotherapy versus anticoagulation).

The case demonstrates that the exclusion of organic causes is just one side of the coin. The other side consists of the search for positive findings. Positive neurological signs, i.e. inconsistencies of the neurological examination (Criterion C in DSM-5) were negative. Here, it has to be considered that the rather low sensitivity of sensory signs results in limitations of diagnostic certainty [19]. According to ICD-10 criteria, associated psychological stress and conflicts were sought for specifically. Although the patient’s history with its many strains might have suggested psychosocial stress at first sight, an experienced psychosomatic clinician finally concluded that there was not sufficient evidence supporting an immediate link between the patient’s history and the onset of his neurological symptoms. Here it is important, not to rely on psychological explanations too quickly. To discern the importance of life events is certainly a difficult task, which can be operationalized only to a limited part (see also [11]).

From a comprehensive clinical point of view, we want to emphasize that in patients who do suffer from a functional neurological disorder, the treatment process, i.e. the integration of a psychosocial understanding of the symptoms, has to begin *parallel to* the diagnostic work-up. These patients are often reluctant to consider psychosocial issues. This applies to “somatoform patients” in general - often resulting in unnecessary and costly diagnostic work-up, which perpetuates the symptom duration [8].

From our point of view and in agreement with the literature [3], well-trained psychosomatic physicians or psychologists should be involved in such cases. In Germany, a special certified training board for psychosomatic medicine and psychotherapy exists since 2003, involving psycho-diagnostic, psychotherapeutic, medical and psychiatric training. In this context the notion of Stone and colleagues that functional neurological symptom disorder is falling into “no man’s land between neurology and psychiatry” is of much interest [2,14]. This has also been emphasized by others [3]. Due to the high specialization and the separation of neurology and psychiatry, patients suffering from functional neurological symptom disorder profit in particular from a psychosomatic consultation and liaison service. The psychosomatic assessment extends the descriptive diagnoses according to ICD and DSM by focusing on issues of psychosocial development and adaptation such as emotion regulation, attachment, etc. With respect to the etiology of functional neurological symptom disorder, multiple influences of different weight are to be considered, including conditioning processes, object relations and identification processes, narcissistic regulation, unconscious (libidinous) strives and conflicts, trauma and personality traits, as well as the social context [12,13,23]. In this context, the “Operationalized Psychodynamic Diagnosis, OPD-2” is of help for the diagnostic and treatment formulation [24]. Another helpful diagnostic tool is the Diagnostic Criteria for Psychosomatic Research (DCPR) [25]. The issue of a wider biopsychosocial assessment also reflects the divergence of “conversion entities” and their various etiologies: These extend from minor and transient conditions of psychological distress, which might require only reassurance and support, to complex traumatized patients with various co-morbidities, who may require long-term psychotherapy and psychopharmacology.

Finally, a few general aspects should be considered: Within the context of diagnostic work-up, positive and negative predictive values (as well as sensitivities and specificities) are never 100%. Therefore, all clinical judgments are approximations. This apparently simple fact is often forgotten too readily. In this context, our basic clinical and scientific attitudes are challenged, which has recently been discussed in the context of DSM-5 by Sisti et al. in this journal [9]. Furthermore, to narrow the focus on biological constructs does not serve scientific success, neither does any other restrictive view; instead “most researchers understand science and medicine to be a social enterprise, shot through with values be they molecular discoveries or clinical breakthroughs” [9]. A biopsychosocial attitude as discussed by Engel can best serve science and clinical work [26]. This will also help medical specialties to better understand each other's language [27]. With respect to discussions of ICD-11 criteria for functional neurological symptom disorder, one might consider the following: Positive psychological or neurological signs are *not the sine qua non* for the diagnosis but they increase the likelihood of the disorder – similar to McDonald criteria for encephalomyelitis disseminata – which is revised according to new empirical evidence.

It was not the purpose and not in the scope of this report to go further into the interesting issues of functional and structural cerebral abnormalities in functional neurological symptom disorder or the important clinical topic of “stroke mimics” [28,29]. However, with respect to the former, Freud - a neurologist/neuropathologist by origin - stated that psychoanalysis is a framework for therapy - the biological basis of it not being known at his time [30]. Neither was it the intention of this report to consider co-morbidity or outcome issues of conversion disorder [18,31]. However, not only is evidence concerning diagnostic criteria limited, but there are no randomized controlled trials with respect to (psycho-) therapy of conversion disorder, though a recent meta-analysis for somatoform disorders gives some hope [32]. Disorder-specific interventions and therapeutic attitudes seem of relevance [33].

## Conclusion

The case presented emphasizes issues of assessment and diagnosis in functional neurological symptom disorder. The value of a close interdisciplinary work-up and cooperation between neurology and psychosomatic medicine is illustrated. Furthermore, a thorough psychosomatic evaluation with respect to treatment planning is essential. Future studies on the validity of positive signs (neurological and psychological) of functional neurological symptom disorder, which is of high clinical relevance [23], are needed. With respect to ICD-11, we recommend to discuss positive psychological as well as positive neurological clues - maybe by increasing its likelihood in a stepwise pattern - in addition to excluding organic disease.

### Patient consent

Written informed consent was obtained from the patient for publication of this case report.

## Competing interests

The authors declare that they have no competing interests.

## Authors’ contributions

CES and AJ planned the report. KB, SR and MK treated the patient. All authors contributed to the writing and approved the manuscript.

## Pre-publication history

The pre-publication history for this paper can be accessed here:

http://www.biomedcentral.com/1471-244X/14/158/prepub
